# Oil spill identification in X-band marine radar image using K-means and texture feature

**DOI:** 10.7717/peerj-cs.1133

**Published:** 2022-10-24

**Authors:** Rong Chen, Bo Li, Baozhu Jia, Jin Xu, Long Ma, Hongbo Yang, Haixia Wang

**Affiliations:** 1 Naval Architecture and Shipping College, Guangdong Ocean University, Zhanjiang, Guangdong, China; 2 Technical Research Center for Ship Intelligence and Safety Engineering of Guangdong Province, Guangdong, China; 3 Navigation College, Dalian Martime University, Dalian, Liaoning, China

**Keywords:** Oil spill extraction, GLCM, Texture feature, K-means, Local adaptive threshold

## Abstract

Marine oil pollution poses a serious threat to the marine ecological balance. It is of great significance to develop rapid and efficient oil spill detection methods for the mitigation of marine oil spill pollution and the restoration of the marine ecological environment. X-band marine radar is one of the important monitoring devices, in this article, we perform the digital X-band radar image by “Sperry Marine” radar system for an oil film extraction experiment. First, the de-noised image was obtained by preprocessing the original image in the Cartesian coordinate system. Second, it was cut into slices. Third, the texture features of the slices were calculated based on the gray-level co-occurrence matrix (GLCM) and K-means method to extract the rough oil spill regions. Finally, the oil spill regions were segmented using the Sauvola threshold algorithm. The experimental results indicate that this study provides a scientific method for the research of oil film extraction. Compared with other methods of oil spill extraction in X-band single-polarization marine radar images, the proposed technology is more intelligent, and it can provide technical support for marine oil spill emergency response in the future.

## Introduction

With the development of maritime shipping, oil pipeline transportation, and drilling platform, the frequent occurrence of oil spills brought about by the increase of offshore transportation accidents and the enhancement of offshore oil and gas resources development capacity is one of the important threats to marine ecological safety. Therefore, the rapid and effective extraction of the location of oil film and its drifting and spreading range has become an important prerequisite for oil spill management (Sun et al., 2013).

Remote sensing is the most common way to monitor oil spills, which mainly includes optical remote sensing and microwave remote sensing. There are many studies of oil spill monitoring methods based on satellite remote sensing data such as Modis, NOAA, and LANDSAT (Hu et al., 2003; Casciello et al., 2011; Taravat & Frate, 2012). These optical sensors have the advantages of a wide monitoring range, low capital investment, high timeliness, and rich spectral information, and have become an important technical means for marine oil spill detection. However, owing to the limitation of spatial resolution, these types of sensors are not capable of accurate extraction of small-scale oil spill regions (Liu et al., 2017). Synthetic Aperture Radar (SAR) is an active microwave high-spatial resolution imaging sensor, the SAR revisit time may be critical but new technologies and new SAR constellations can mitigate such a problem (Mdakane & Kleynhans, 2022). Polarimetric SAR observations lead to a significant improvement in sea oil slick observation since they allow distinguishing oil slicks from a broad class of lookalikes in an unsupervised way (Migliaccio, Nunziata & Buono, 2015). Using X-band dual-polarization co-polarization SAR images, the effects of imaging parameters and environmental conditions on oil spill observation were comprehensively analyzed to study the oil spill area for a long time series (Nunziata et al., 2019). Comparative analysis of C-band and X-band SAR data for marine oil spill observation using statistical properties and selected multi-polarization (HH, VV) parameters (Skrunes et al., 2015). Considering the performance of amplitude coherence and co-polarization phase difference (CPD) standard deviation in offshore oil slick observation, a study of offshore oil observation using dual-polarized X-band SAR data was carried out, and the results showed the advantages of the CPD method and the effectiveness of TerraSAR-X dual-polarization products in oil spill monitoring applications (Lehner et al., 2011). With the continuing advances in information technology, airborne oil spill monitoring based on optical cameras, video cameras, and infrared sensors began to develop (Sudhir et al., 2008; Liu, Li & Gao, 2014; Vagata, Pinho & Hengstermann, 2016). Airborne detection has the advantage of high flexibility, and it can continuously monitor oil film variation over a period of time (Collins et al., 2015). However, the monitor range is limited and greatly affected by illumination and meteorological conditions (Eliza et al., 2011). Marine radar, also known as navigation radar, can cooperate with vessels to clean up the oil spills and obtain the oil spill pollution regions within a certain range around the ship in an all-weather, real-time, and efficient manner. It can overcome certain harsh sea conditions and carry out oil spill monitoring emergency treatment on the ship, which has significant prospects for application and plays an important role in oil spill monitoring (Wang, Liu & Cheng, 2017; Xu et al., 2020; Zhao et al., 2020). In 1971, the United States first used marine radar to observe oil spill in the Gulf of California, after that, the research on marine radar oil spill monitoring began to increase. The oil spill monitoring capability of marine radar was evaluated during a cruise off the coast of Nova Scotia, Canada (Tennyson, 1988). The X-band marine radar tracked and recorded the oil spill along the Black Sea coast, and various parameters describing the characteristics of the oil spill were evaluated (Atanassov et al., 2002). At present, there are a few studies on oil spill extraction by shipboard radar images. A texture feature analysis method that used a marine radar image as input has been proposed, in which the oil film was accurately extracted by a threshold segmentation algorithm (Liu et al., 2019). However, the whole image is traversed and classified using a sliding window; although the oil film extraction accuracy is very high, the amount of calculation is very large. Another method of analyzing a marine radar image used the Otsu algorithm and obtained the region of the oil spill (Zhu, Li & Liu, 2015). This method is simple and it can quickly identify oil spill targets and false positive targets. However, the algorithm is based on a global threshold, and it is greatly affected by illumination, which may cause inaccurate segmentation.

With the development of image processing technology, machine learning has been well applied in oil spill image classification and target extraction. Applications such as K-means, support vector machine (SVM), K-nearest neighbor (KNN), artificial neural network (ANN), and convolutional neural network (CNN) have been used in testing oil films, and the results showed that the oil film area can be accurately predicted by machine learning (Li et al., 2021). Based on the multi-polarization characteristics of the SAR image and the K-means algorithm, the oil film was extracted. It was proved that the feature-based K-means classification is considered to be at least as good as the standard Wishart clustering of the covariance matrix (Skrunes, Brekke & Eltoft, 2014). A modified K-means clustering was used to detect and segment the oil spill in the ocean (Ganesan, 2015). By using a combination of LBP and K-means, the oil spill extraction experiment was operated successfully (Xu et al., 2021a; Xu et al., 2021b). Two different artificial neural networks were employed to detect oil spills of SAR images which classified objects into oil spills or look-alikes (Singha, Bellerby & Trieschmann, 2013). Employed by the convolutional neural network method and infrared imaging camera, an oil spill accident at night was detected (Kerf et al., 2020). A deep convolutional neural network was used for oil spill detection from SAR Image, the classification performance of which was significantly improved compared to that of traditional machine learning (Zeng & Wang, 2020). Based on the polarization decomposition characteristics of the SAR image, a support vector machine was employed for oil spill detection (Zou et al., 2016). A novel method was proposed to discriminate different kinds of spilled oil, which was the qualitative analysis model based on the support vector machine and can work for rapid identification of spilled oil (Tang, Bi & Zhao, 2011). Based on multispectral satellite data, the K-nearest neighbor was used to classify the oil image objects to monitor large oil slick dynamics (Pieralberto et al., 2014). By adopting object-based classification KNN and visual interpretation, the semi-automatic detection and discrimination of oil spills, and natural seepage slicks were tested in the Caspian region (Emil, Martin & Manfred, 2018). Also, a multi-class neural network was used to monitor an oil spill at sea and improved the oil pollution cleaning method, which is of great significance to preventing and controlling marine pollution (Ghorbani & Behzadan, 2021).

In recent years, many oil spill identification studies are about SAR and multispectral visible light images, while there are fewer reports on marine radar oil spill extraction. One of the important reasons is that the number of marine radar oil spill images is limited. Marine radar oil spill monitoring is an important means, based on the sea clutter characteristics, the ability of marine radar to monitor oil spills is because when the sea surface is covered with an oil film, the oil film can suppress capillary waves and make the seawater surface smoother, thus reducing the backward scattering intensity of radar waves, resulting in a lower gray value on the radar image and forming a dark area on the image that is significantly darker than the surrounding environment. Although the backscatter echo signal varies with the oil type, it is reflected in the image as a dark target, but with different intensities. Meanwhile, marine radar can carry out real-time online monitoring and alarm for all-weather oil spills, and the application of oil spill identification technology to shipboard radar can facilitate emergency response departments. What needs to be clear here is that the emergency treatment of oil spill accidents emphasizes fast and efficiency, and it needs to identify and extract oil film in a way with universality and fast operation speed. Since the radar image is a grayscale image, it is not convincing to identify oil film and non-oil film only by the difference in gray-scale values. So, texture features need to be introduced to reduce the identification error. Considering the accident in our study is crude oil spills, we use threshold segmentation based on the gray value of the image. In these contexts, this article proposes an intelligent identification method for oil spills of marine radar data. Based on comparisons of texture features, local adaptive thresholds, oil spill identification methods, slice window sizes, and machine learning classifiers, an oil spill film identification method combining the texture feature of GLCM theory and the K-means clustering algorithm is proposed, which provides an approach for oil spill extraction from marine radar images.

## Materials & Methods

### Study area and experimental data

#### Study area

On July 16, 2010, a CNPC oil pipeline near Dalian Xingang, China, caught fire and exploded, causing a crude oil leak. As a result, at least 50 square kilometers of the nearby sea were polluted by crude oil. Although the departments took immediate emergency response measures, the uncertainty of the crude oil drifting and spreading on the sea surface brought pressure and challenges to the clean-up work. After the oil spill accident, we conducted oil spill photography through marine radar. Through experiments, we proposed an efficient and intelligent oil spill identification method, thus facilitating future emergency response work on the sea.

#### Experimental data

**Figure 1:**
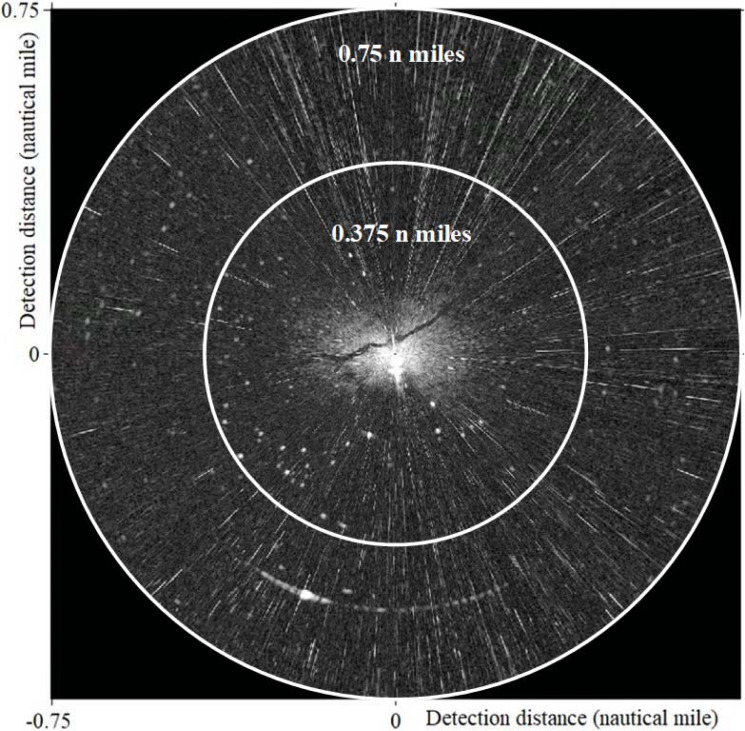
Experimental data.

In our study, the “Sperry Marine” radar system was used to monitor and record the wave clutter signals. The signals output from the radar transceiver was directly connected to the computer processing system, and the image was displayed by the monitor after processing. With the rotation of the radar antenna, the radar system can digitize and store radar images of the sea surface. Table 1 displays the main parameters of radar. In this work, the experimental data is the X-band horizontal polarization image collected at 23:19 on July 21, 2010, by the teaching-practice ship *Yukun* of Dalian Maritime University (Fig. 1). The image size was 1,024 × 1,024 pixels with a detection range of 0.75 nautical miles (NM), and the actual area represented by each pixel is 7.36 m^2^.

**Table 1:** The parameters of radar.

Parameter	Value
Band	X-band
Detection distance	0.5/0.75/1.5/3/6/12/24 NMs
Range resolution	3.75m
Antenna type	Waveguide split antenna
Polarization mode	Horizontal
Horizontal detection angle	360°
Rotation speed	28–45 revolutions/min
Length of antenna	8ft
Pulse recurrence frequency	3,000 Hz/800 Hz/785 Hz
Pulse width	50n/ns/ns
Automatic image acquisition speed	28 images/min
Spatial resolution	7.36 m^2^

#### Data preprocess

**Figure 2:**
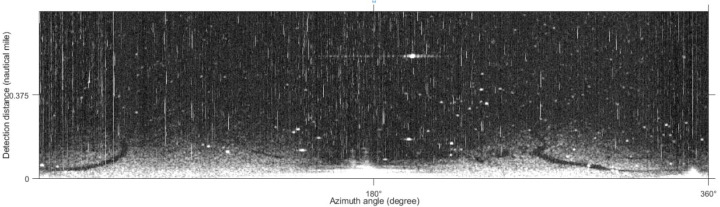
Original radar image.

**Figure 3:**
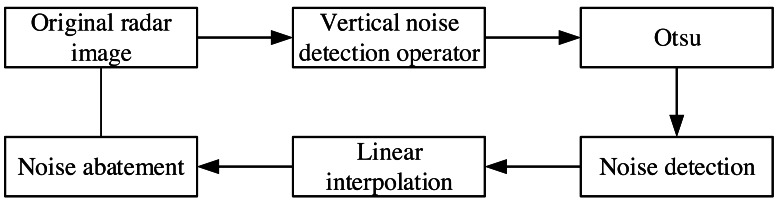
Preprocess scheme.

**Figure 4:**
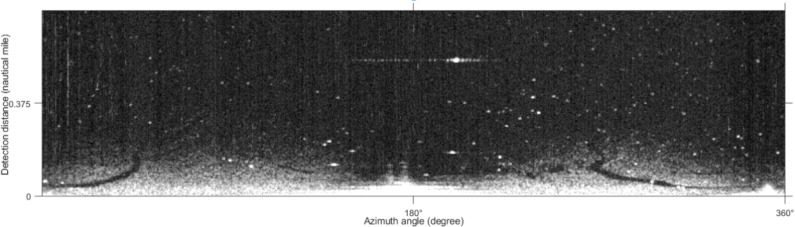
De-noised image.

The collected experimental image adopts the polar coordinate system, which needs to be converted into the Cartesian coordinate system to facilitate subsequent processing. The image after the coordinate transformation takes the azimuth angle as the horizontal axis and distance as the vertical axis. The image size is 512 × 2,048 pixels (Fig. 2). The bright line in Fig. 2 is the co-frequency vertical interference noise, and the lighter-colored areas at the bottom of the image are wave echoes and oil film targets. The original image needs to be preprocessed to extract the oil film region successfully. The pretreatment process referred to the methods adopted by Xu et al. (2021a); Xu et al. (2021b), and the specific process is shown in Fig. 3. First, the vertical noise detection operator is used to convolve with the image in the Cartesian coordinate system. Second, the Otsu algorithm is used for detecting vertical noise processing. Third, the distance weighted linear interpolation method is used to suppress the vertical noise. Finally, the de-noised image is obtained (Fig. 4). In this article, the method of oil film extraction was studied using the de-noised image and based on the Matlab R2020b platform.

### Experimental method

**Figure 5:**
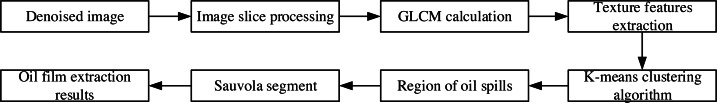
Process scheme.

In order to realize an intelligent, and rapid marine oil spill monitoring method, effectively improve the efficiency of oil spill monitoring, we compared and tried to select a feature in GLCM with typical machine learning classification methods, and then it combined with the local adaptive threshold to effectively extract oil film information from the marine radar image. The experiment process is shown in Fig. 5. First, the denoised image was cut into slices the size of the local window. Second, the texture features of each slice were calculated based on the GLCM. Third, according to the texture features, the K-means clustering algorithm was used to extract the effective oil spill area from the cut images. Finally, using the Sauvola algorithm, the oil film was segmented, and the final extracted oil film region was overlapped on the image.

### Texture feature extraction based on the GLCM

**Figure 6:**
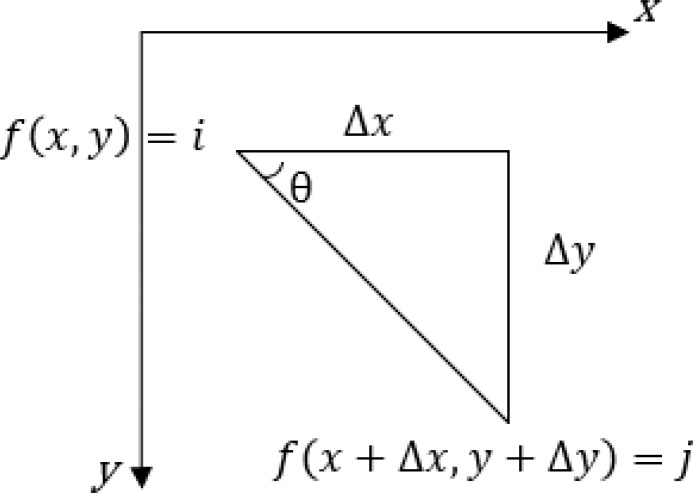
The mechanism of GLCM.

The texture is caused by the different physical attributes of the object surface and mainly reflects the diversity of grayscale or color information. Image texture is one of the attributes of images, which usually be represented by the gray distribution of a pixel and its surrounding spatial neighborhood (Mohanaiah, Sathyanarayana & Gurukumar, 2013). GLCM is a common method to describe texture by studying the spatial correlation characteristics of gray. It is defined as the probability that two pixels with step distance d and direction *θ* appear in the image (Barburiceanu, Terebes & Meza, 2021), which is expressed as formula Eq. (1), the mechanism of GLCM is shown in Fig. 6. Through the GLCM, the image texture features can be extracted, and Table 2 lists the calculation formula of each texture feature characterization quantity.

(1) \begin{eqnarray*}{p}_{ij}= \frac{p(i,j,d,\theta )}{\sum _{i=0}^{M}\sum _{j=0}^{N}p(i,j,d,\theta )} \end{eqnarray*}

**Table 2:** Texture features formula.

Texture feature	Formula
Angular second moment	${f}_{ASM}={\mathop{\sum }\nolimits }_{i=0}^{M}{\mathop{\sum }\nolimits }_{j=0}^{N}p(i,j,d,\theta )^{2}$
Entropy	${f}_{ENT}=-{\mathop{\sum }\nolimits }_{i=0}^{M}{\mathop{\sum }\nolimits }_{j=0}^{N}p \left( i,j,d,\theta \right) \log p(i,j,d,\theta )$
Contrast	${f}_{CON}={\mathop{\sum }\nolimits }_{i=0}^{M}{\mathop{\sum }\nolimits }_{j=0}^{N}{ \left( i-j \right) }^{2}p(i,j,d,\theta )$
Mean	${f}_{MEAN}={\mathop{\sum }\nolimits }_{i=0}^{M}{\mathop{\sum }\nolimits }_{j=0}^{N}i\ast p(i,j,d,\theta )$
Homogeneity	${f}_{HOM}={\mathop{\sum }\nolimits }_{i=0}^{M}{\mathop{\sum }\nolimits }_{j=0}^{N} \frac{p(i,j,d,\theta )}{1+(i-j)^{2}} $
Dissimilarity	${f}_{DIS}={\mathop{\sum }\nolimits }_{i=0}^{M}{\mathop{\sum }\nolimits }_{j=0}^{N} \left\vert i-j \right\vert p(i,j,d,\theta )$
Correlation	${f}_{COR}={\mathop{\sum }\nolimits }_{i=0}^{M}{\mathop{\sum }\nolimits }_{j=0}^{N} \frac{ \left( i-\mu \right) \left( j-\mu \right) p(i,j,d,\theta )}{{\sigma }^{2}} $
Variance	${f}_{var}={\mathop{\sum }\nolimits }_{i=0}^{M}{\mathop{\sum }\nolimits }_{j=0}^{N}{ \left( i-\mu \right) }^{2}p(i,j,d,\theta )$

**Notes.**

$\mu ={\mathop{\sum }\nolimits }_{i=0}^{M}{\mathop{\sum }\nolimits }_{j=0}^{N}i\times p(i,j,d,\theta )$, ${\sigma }^{2}={\mathop{\sum }\nolimits }_{i=0}^{M}{\mathop{\sum }\nolimits }_{j=0}^{N}{ \left( i-\mu \right) }^{2}p(i,j,d,\theta )$, where µrepresents mean, *σ*^2^ represents variance.

### K-means clustering algorithm

The K-means clustering algorithm is an iterative algorithm based on unsupervised learning. It is widely used in data classification because of its simple realization, strong explanatory power, and effective clustering effect (Nitta et al., 2020). The algorithm process is as follows (Hartigan & Wong, 1979): (1) K initial cluster center *c*_*i*_ ( *i* = 1, 2, 3,…,K) is selected for the sample set S, where S = {*x*_1_, *x*_2_ ,…, *x*_*n*_}. (2) The Euclidean distance between each object and K cluster centers is calculated, and the data object to the cluster that is closest to the cluster center is classified. (3) The mean value of data objects in each cluster is calculated, and the mean value is taken as the new cluster center.

(2) \begin{eqnarray*}{c}_{i}= \frac{1}{ \left\vert {S}_{i} \right\vert } \sum _{{x}_{j}\in {x}_{i}}{X}_{j}\end{eqnarray*}

where $ \left\vert {S}_{i} \right\vert $ isthe total number of instances that are in cluster *i*.

(4) The distance of each data object to the new K initialization cluster centers is calculated and redivided.

(5) The next iteration proceeds until the object category stops changing and the clustering ends.

### Local adaptive threshold segmentation algorithm

The local adaptive threshold segmentation algorithm is based on the distribution of pixel gray values in the window. The gray mean *s*(*i*, *j*) and gray standard deviation $m \left( i,j \right) $ were used to calculate the threshold $T \left( i,j \right) $. Compared with global thresholds, local adaptive thresholds can avoid segmenting the noise in images as well as deal with the problem of low-resolution images.

Niblack (1986) proposed a local threshold method for the image pixel-level process. This process involves adjacent pixel values within a region window. The threshold T(i,j) can be estimated as:

(3) \begin{eqnarray*}T \left( i,j \right) =m \left( i,j \right) +ks(i,j)\end{eqnarray*}

where *k* is a constant.

Sauvola & Pietikinen (2000) improved on the Niblack algorithm. Its expression is as follows:

(4) \begin{eqnarray*}T(i,j)=m(i,j)\times \left[ 1+k \left( \frac{s \left( i,j \right) }{R} -1 \right) \right] \end{eqnarray*}

where *R* is the dynamic range of standard deviations, and *k* is the influence factor of standard deviation, which reflects the intensity of the influence of standard deviation on the threshold *T*(*i*, *j*). The range is between 0 and 1.

Phansalkar et al. (2011) modified the Sauvola local adaptive threshold segmentation algorithm, which is used for processing low-contrast images. $T \left( i,j \right) $ can be expressed as follows:

(5) \begin{eqnarray*}T \left( i,j \right) =m \left( i,j \right) \left[ 1+p{e}^{-q\cdot m \left( i,j \right) }+k \left( \frac{s \left( i,j \right) }{R} -1 \right) \right] \end{eqnarray*}

where *p* and *q* are constants.

After comparing the experiments, the Sauvola algorithm was chosen for oil film identification in our work.

## Result

### Image slices

The size of the each slice is generally the common factor of the length and width of the image, and the commonly used sizes are 8 × 8 pixels, 16 × 16 pixels, 32 × 32 pixels, 64 × 64 pixels, 128 × 128 pixels, and 256 × 256 pixels (Xu et al., 2021a; Xu et al., 2021b). Considering the accuracy and efficiency, In this article, the de-noised image is cut into 64 × 64 pixels and 256 sub-images are generated.

### Texture feature extraction and selection

According to the definition of the GLCM model and the requirements of eigenvalue calculation, parameters of the texture feature extraction algorithm based on the GLCM are selected as follows:

(1) Selection of gray level.

The commonly used gray levels are 16, 64, 128, and 256. To reduce the amount of computation, in our work 16 is chosen as the gray level.

(2) Selection of step length.

The step size adopted in this article was *d* = 1, that is, the central pixel and its adjacent pixels were calculated.

(3) Selection of direction.

Generally, *θ* is 0°, 45°, 90°, and 135°. However, considering that the differences between the four directions are not obvious, this article takes the average values of these four directions.

**Figure 7:**
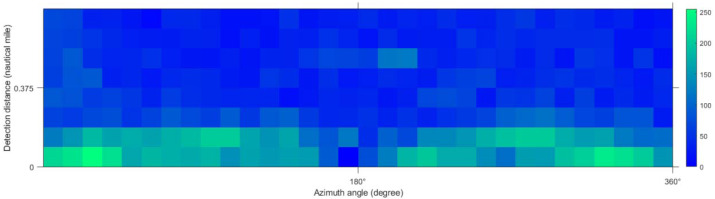
Entropy index value of local window.

According to the formula in Table 2, the characteristic values of each texture were calculated for the de-noised image. GLCM was used to extract texture features of each slice. In this article, texture feature entropy was selected as the classification feature. Figure 7 shows the output result of the entropy value.

### Image classification

**Figure 8:**
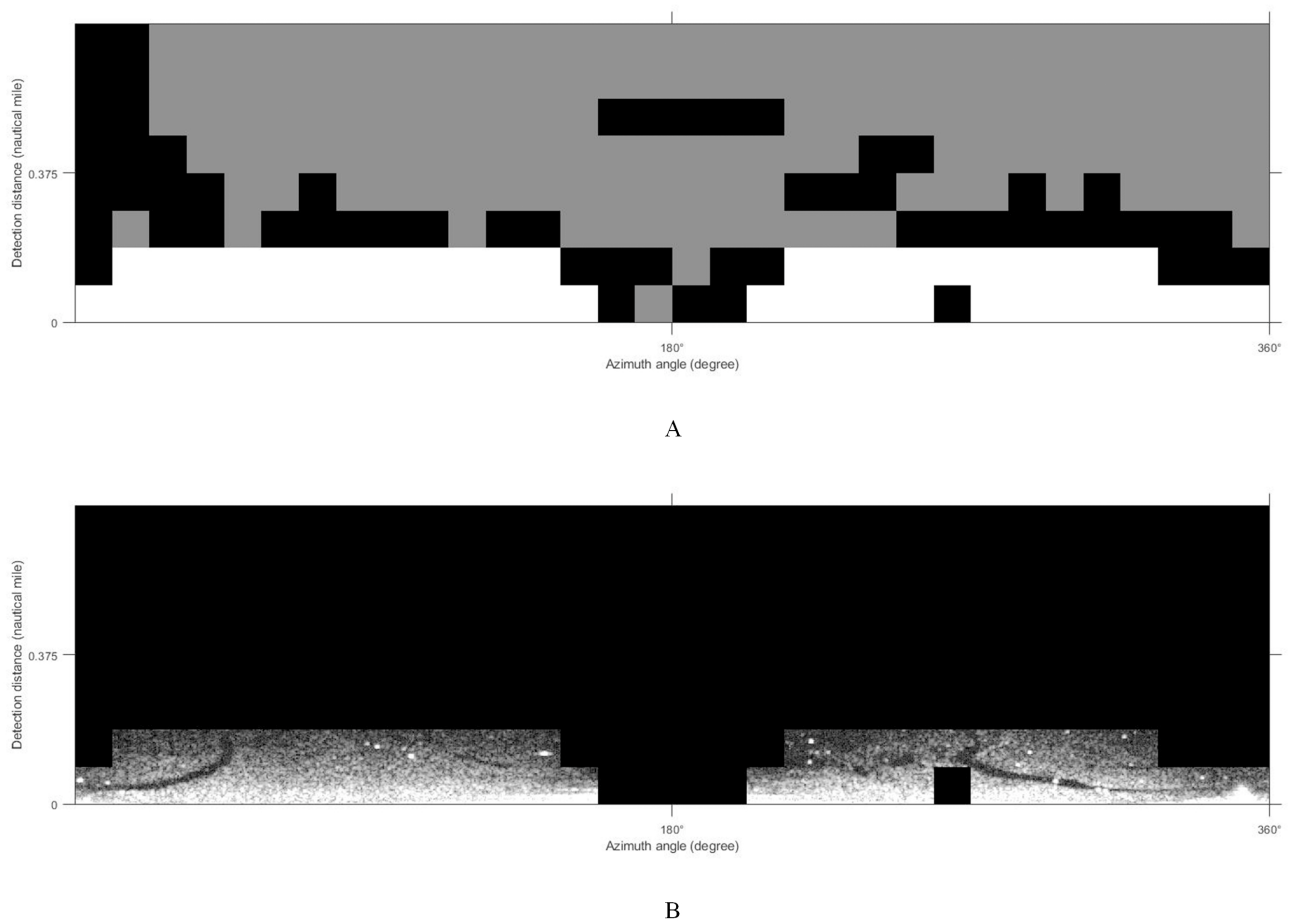
K-means clustering process. (A) Classification results. (B) Classification results on de-noised image.

The K-means clustering algorithm was adopted to classify the sliced images. The final clustering result of this algorithm depends on the arbitrary selection of the cluster center and the size of the *K* value. Different texture features have different abilities to identify targets. In our work, texture feature entropy was taken as the input feature of the classifier. In general, marine radar images mainly record the regions with valid waves, weak wave echo signals, and wave disturbance (Liu et al., 2021). To classify these three regions and lock the oil films position, the initial number of clustering *K* = 3 and the number of iterations 50 were set to classify images. The result of classification is shown in Fig. 8A. Black is the disturbance zone, white is the valid wave area and gray is the region with weak wave echo signals. Considering that the oil films appear on the valid waves, and the wave is at the bottom of the image, the white part of the classification result is retained and superimposed with the de-noised image. The oil film target region is finally generated (Fig. 8B).

### Threshold segmentation

**Figure 9:**
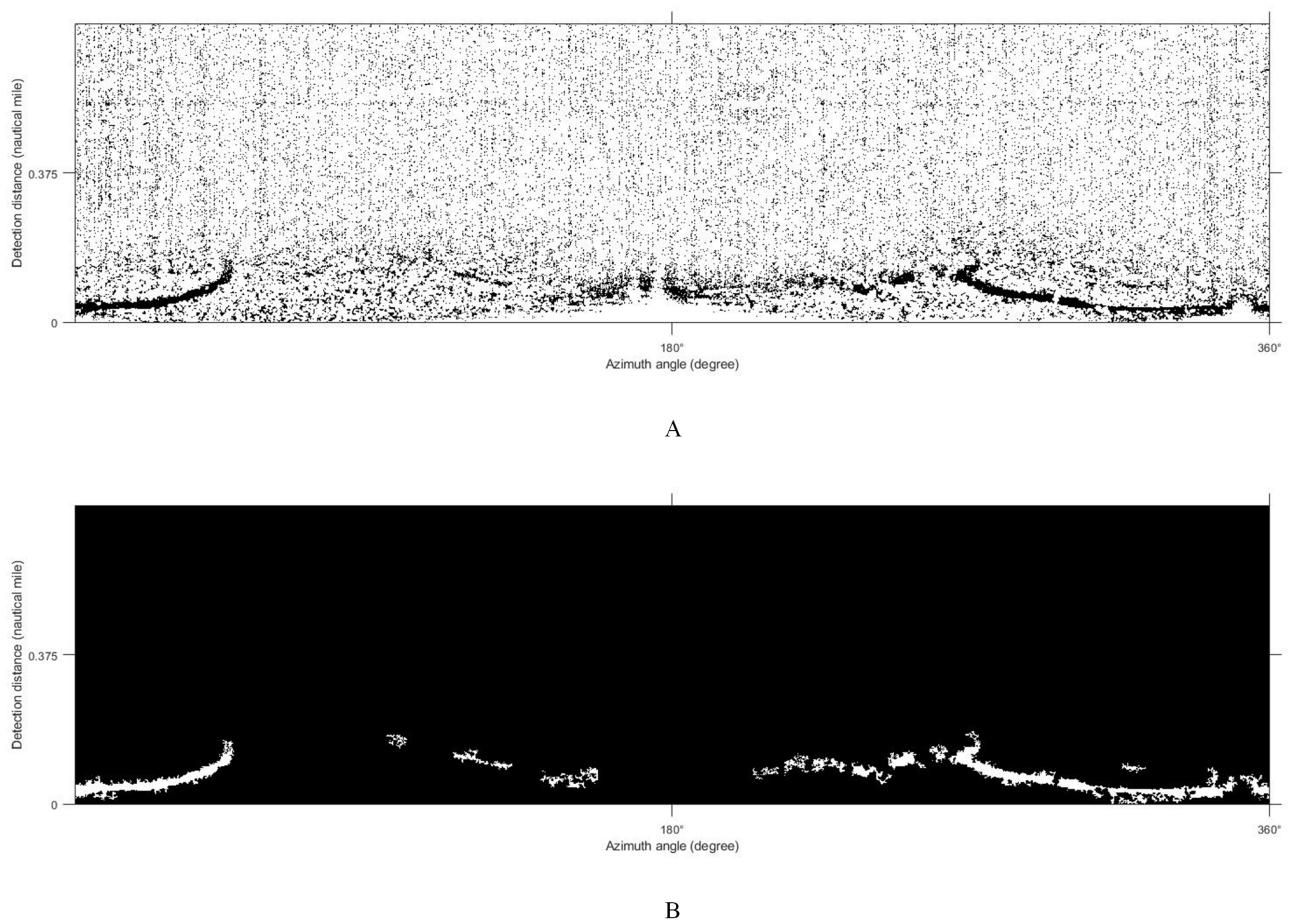
Oil film segmentation process. (A) Segmentation results. (B) Oil film extraction results.

**Figure 10:**
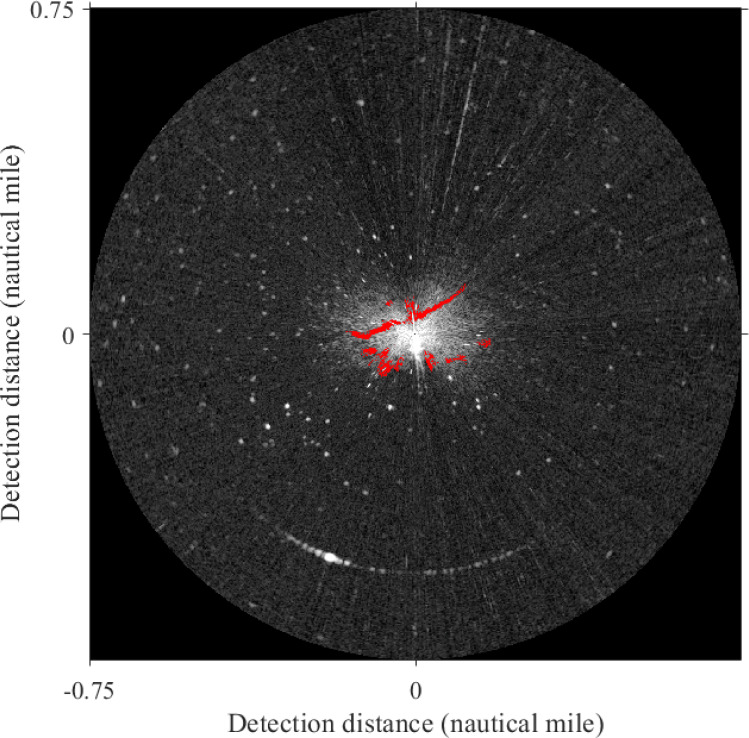
Oil film extraction results in polar coordinate system.

The Sauvola algorithm as used to segment oil spill images, the window size was set to 32 × 32 pixels with the dynamic range of standard deviations *R* = 128 and the influence factor of standard deviation *k* = 0.5. The oil spill image was segmented to obtain the initial resulting image, as shown in Fig. 9A. There are lots of small spots in the preliminary result. Because the oil film is usually continuous, the small areas of black spots were removed. Then the image is inversed, repeating the segmentation operations to remove small areas of white spots. The final oil film extraction result is obtained by superposition with the classification diagram (Fig. 9B). And the image is converted into coordinates. Figure 10 shows the oil spill identification results in the polar coordinate system.

### Validation

**Figure 11:**
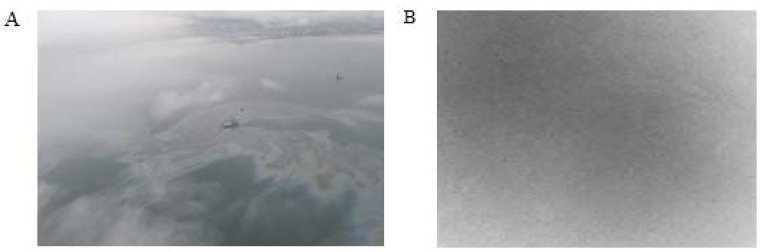
Images of oil spill site. (A) The visible light image. (B) The thermal infrared image.

In our work, the visible light image (Fig. 11A) and thermal infrared image (Fig. 11B) were used to validate the result. Because the collection time of the radar image was at night, it is impossible to capture the same real-phase offshore oil film with the visible sensor. Figure 11A shows the visible image near the acquisition location taken during the daytime, and it is obvious that an oil film exists. Figure 11B shows the oil film captured with the thermal infrared sensor at the same location as in the radar image, and the grayscale value of the area where it is located is slightly lower than the grayscale value of the neighboring area. Meanwhile, some scholars have conducted studies on this oil spill, and eight scenes of remote sensing images of the oil pollution impact area were collected by the HJ1 A/B satellite during the critical period of oil spill response (July 16, 2010–August 2, 2010) (Lan, Ma & Chen, 2012). These images from different sensors proved that oil spill films were present in the sea at that time.

## Discussion

### Comparison of texture features

**Figure 12:**
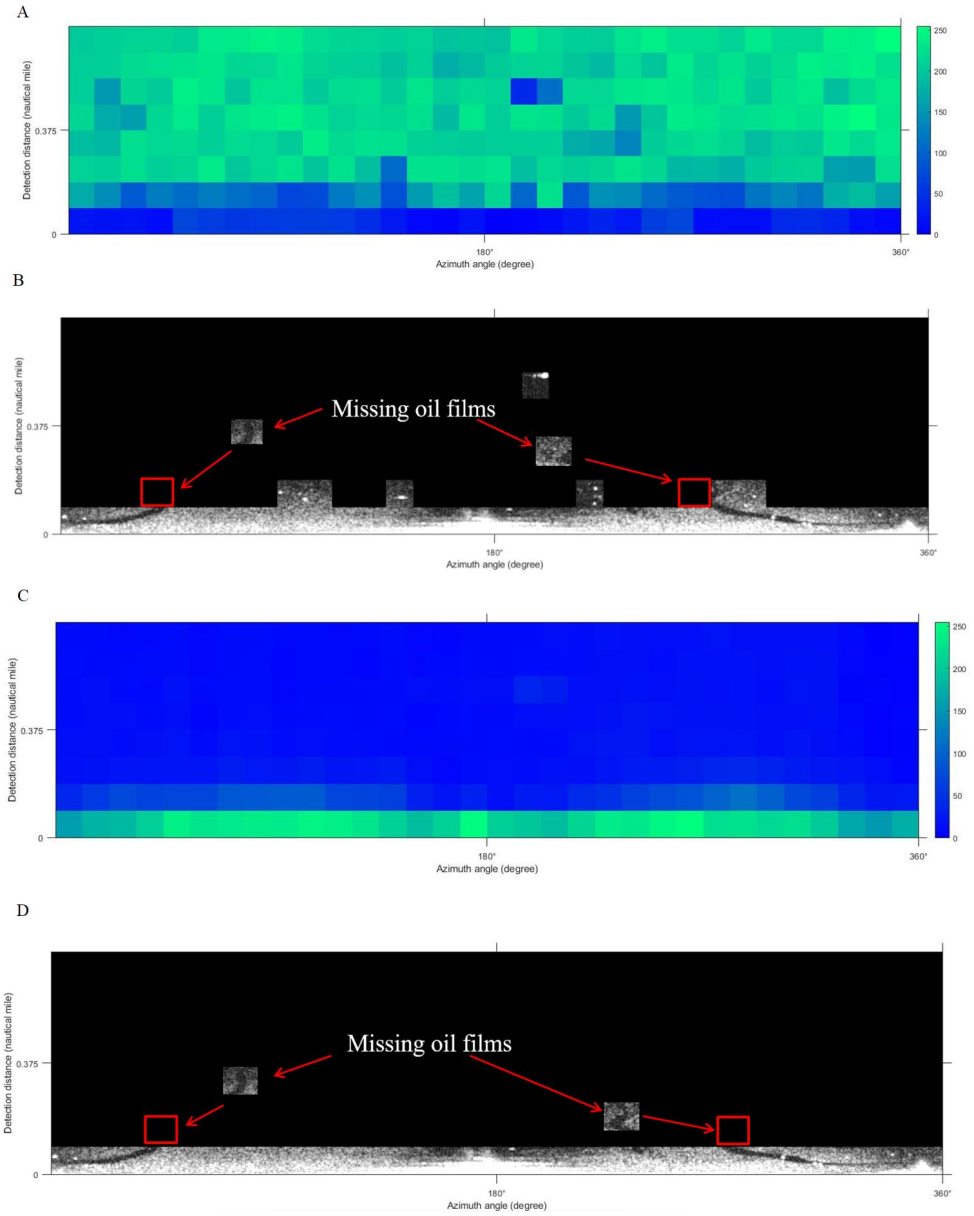
Results based on comparison of texture features. (A) Correlation. (B) Classification result based on correlation. (C) Mean. (D) Classification result based on mean value.

Multiple texture features were extracted using the GLCM method. Ma, Li & Niu (2010) selected texture feature correlation and mean as the texture feature index for oil spill extraction from SAR image. In our work, these two texture features were used as the input to the K-means algorithm to classify the images. Figures 12A and 12C are the visualizations of the texture feature correlation and the mean value. The extracted effective oil spill areas are shown in Figs. 12B and 12D. The results obtained by selecting these two features have the problem of missing oil film, while using the texture feature entropy as the input classifier, the oil film regions are effectively extracted, and the main two strip-shaped oil films remain intact, as shown in Fig. 8B.

### Comparison of local adaptive thresholds

**Figure 13:**
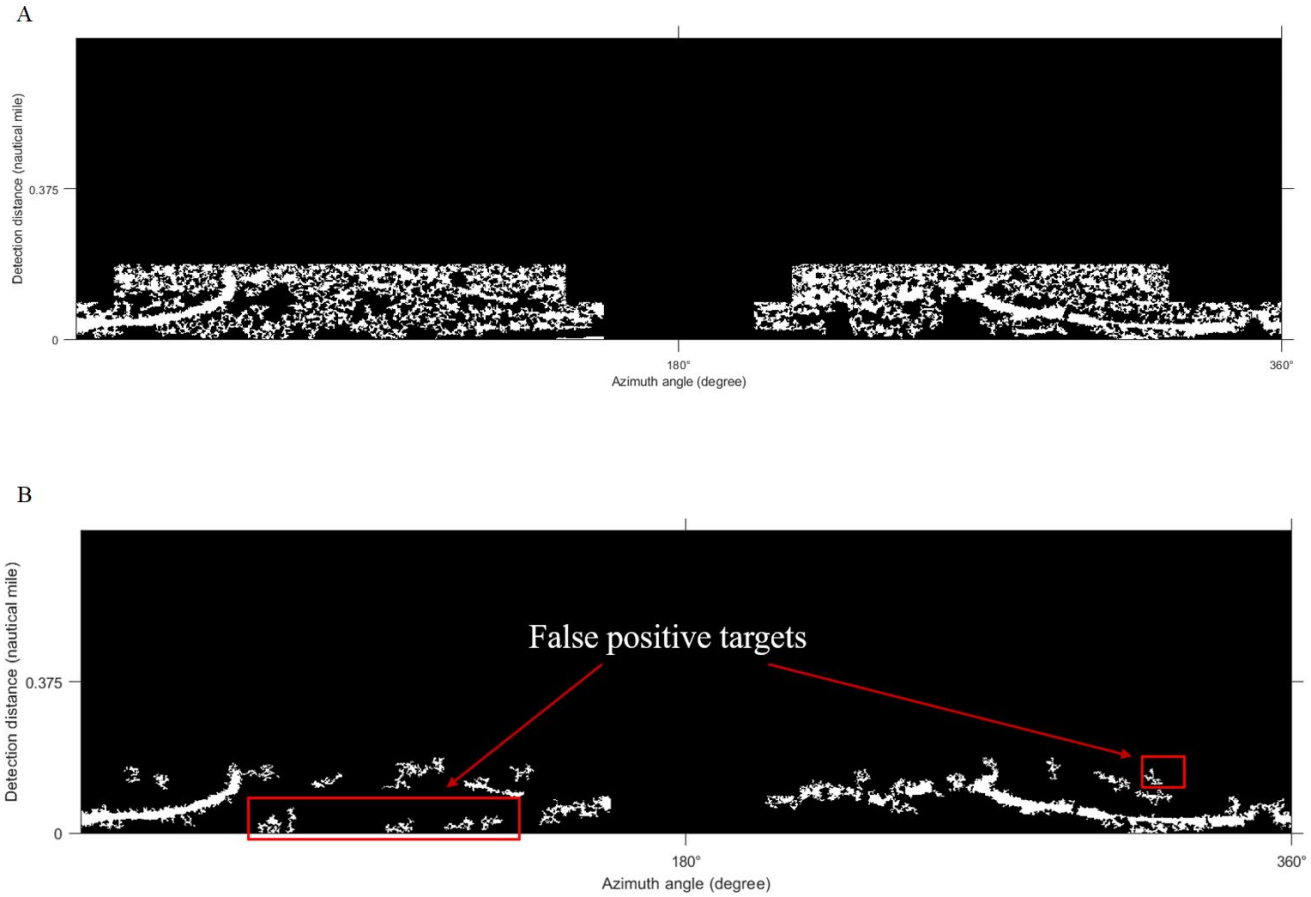
Comparisons of thresholds. (A) Otsu segmentation. (B) Phansalkar segmentation.

**Figure 14:**
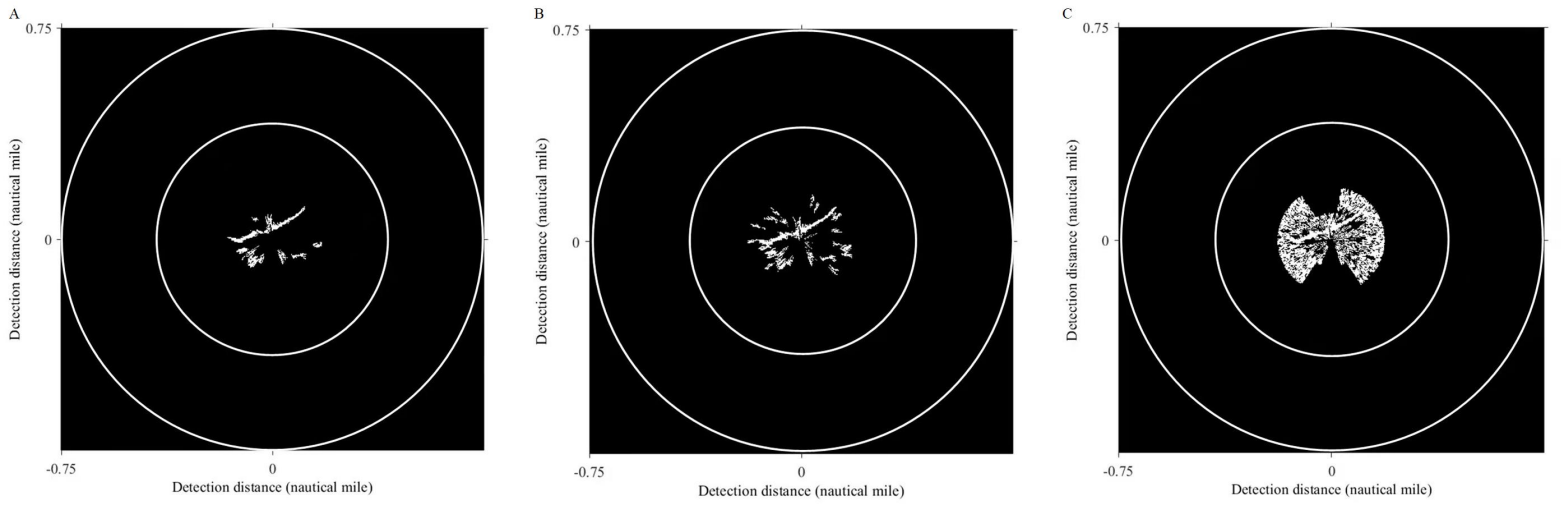
Oil spill identification results from different methods. (A) Sauvola. (B) Phansalker. (C) Otsu.

Xu et al. (2021a); Xu et al. (2021b) used the Phansalkar algorithm for the segmentation of marine radar images to extract oil films. Yu et al. (2017) adopted the improved Otsu algorithm for oil spill detection on SAR images. In our work, the improved Otsu and Phansalkar methods were used to segment the de-noised image to compare with the Sauvola method, the parameters of the Phansalkar method recommend *k* = 0.25, *R* = 0.5, *p* = 2, and *q* = 10, the window size of the Otsu method is set to 128 × 128 pixels., The Otsu algorithm for segmentation could not identify the oil film well, as shown in Fig. 13A. There were many noises mistakenly segmented out. As shown in Fig. 13B, oil film can be extracted by the Phansalkar algorithm. However, some false positive targets were produced. The final oil spill results extracted by the three methods are displayed under the polar coordinate system (Fig. 14), and the oil pixel number and the area of the oil are counted as shown in Table 3. The area calculation shows that the area of the oil film obtained by the Otsu algorithm is much larger than the extraction results of the other two methods, which is caused by a large number of false positive targets being misclassified into oil films. The extracted oil film area by the Phanlakar method is also larger than that of the Sauvola method. Therefore, the proposed Sauvola algorithm is superior to the other two algorithms in segmentation accuracy, and it is suitable for the extraction of marine oil films.

**Table 3:** Pixel number and area of oil films identified under different threshold methods.

Threshold method	Number of oil films pixels	Oil film area (m^2^)
Otsu	23,751	175,282.38
Phansalkar	6,097	44,995.86
Sauvola	3,581	26,427.78

### Comparison with other methods in oil spill identification

**Figure 15:**
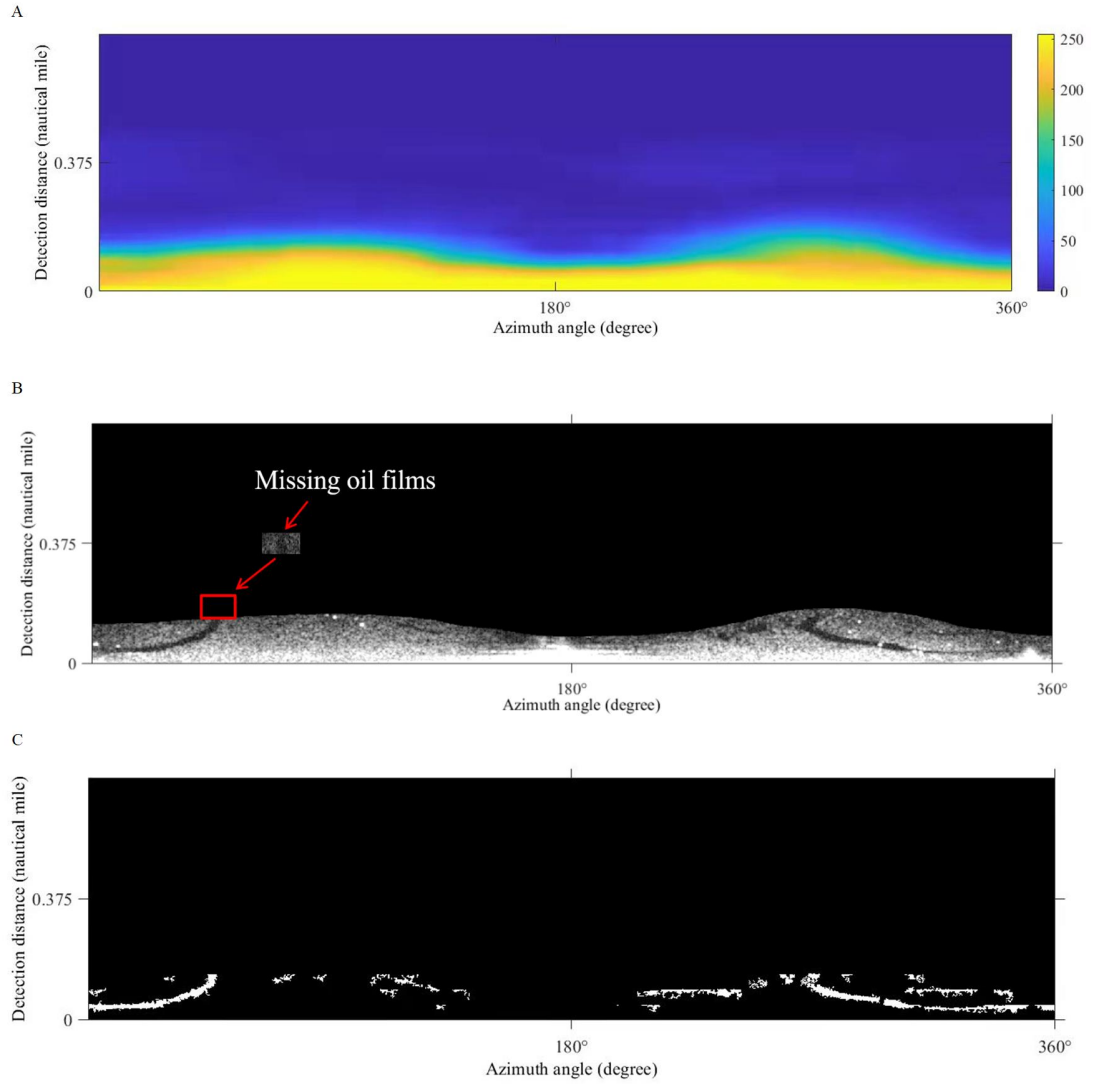
The result of support vector machine and local adaptive threshold method. (A) Gray distribution matrix. (B) The effective wave range. (C) Oil spill identification result.

Xu et al. (2020) adopted the support vector machine and local adaptive threshold method to identify and extract oil spills from shipboard radar (hereafter referred as Method 1). In our work, we use the same method for oil film extraction experiments. First, the support vector machine method is used to distinguish waves from the background. Then the image is processed by image restoration techniques to generate a gray distribution matrix (Fig. 15A). Second, the gray distribution matrix threshold is set to “100” to obtain the effective wave monitoring range (Fig. 15B). Finally, the oil spill identification result is obtained after adaptive threshold segmentation of the effective wave area and removal of small spots (Fig. 15C). Also, in this method, the effective wave region is segmented by manually selecting threshold, which is not an intelligent approach, in other words, the option of threshold value affects the extraction range of the oil film directly.

**Figure 16:**
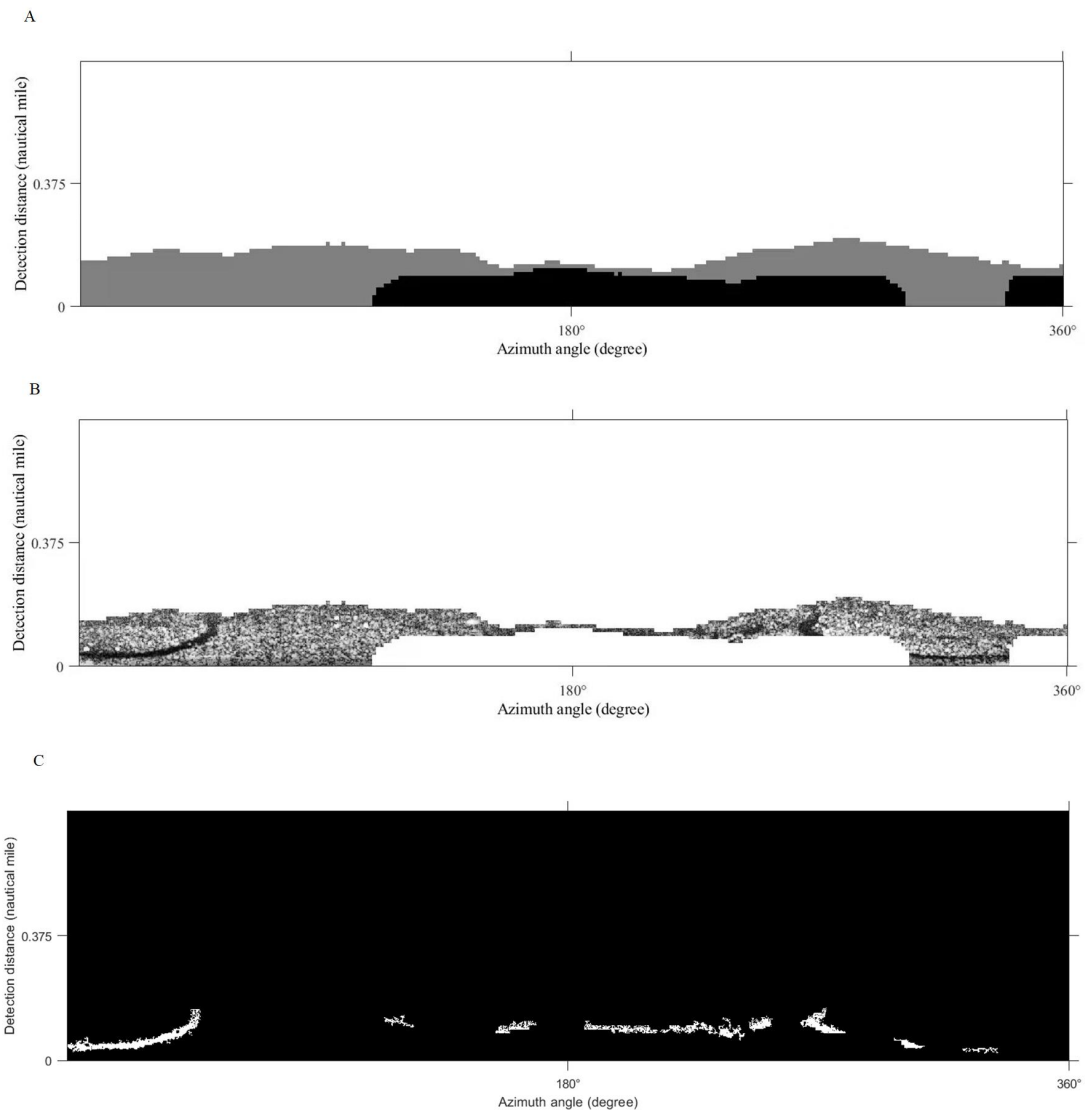
The result of LBP and K-means method. (A) K-means result. (B) The valid wave region.(C) Oil spill identification result.

Xu et al. (2021a); Xu et al. (2021b) conducted an oil spill extraction experiment using a combination of LBP and K-means (hereafter referred as Method 2). Here we use the same method to complete our experiment, the sliding window size is set to 128 pixels. The classification result, the effective oil spill range, and the final oil film identification are shown in Fig. 16, although this method can reject the interference of ship wake flow, there is a problem that many oil films are missing.

**Figure 17:**
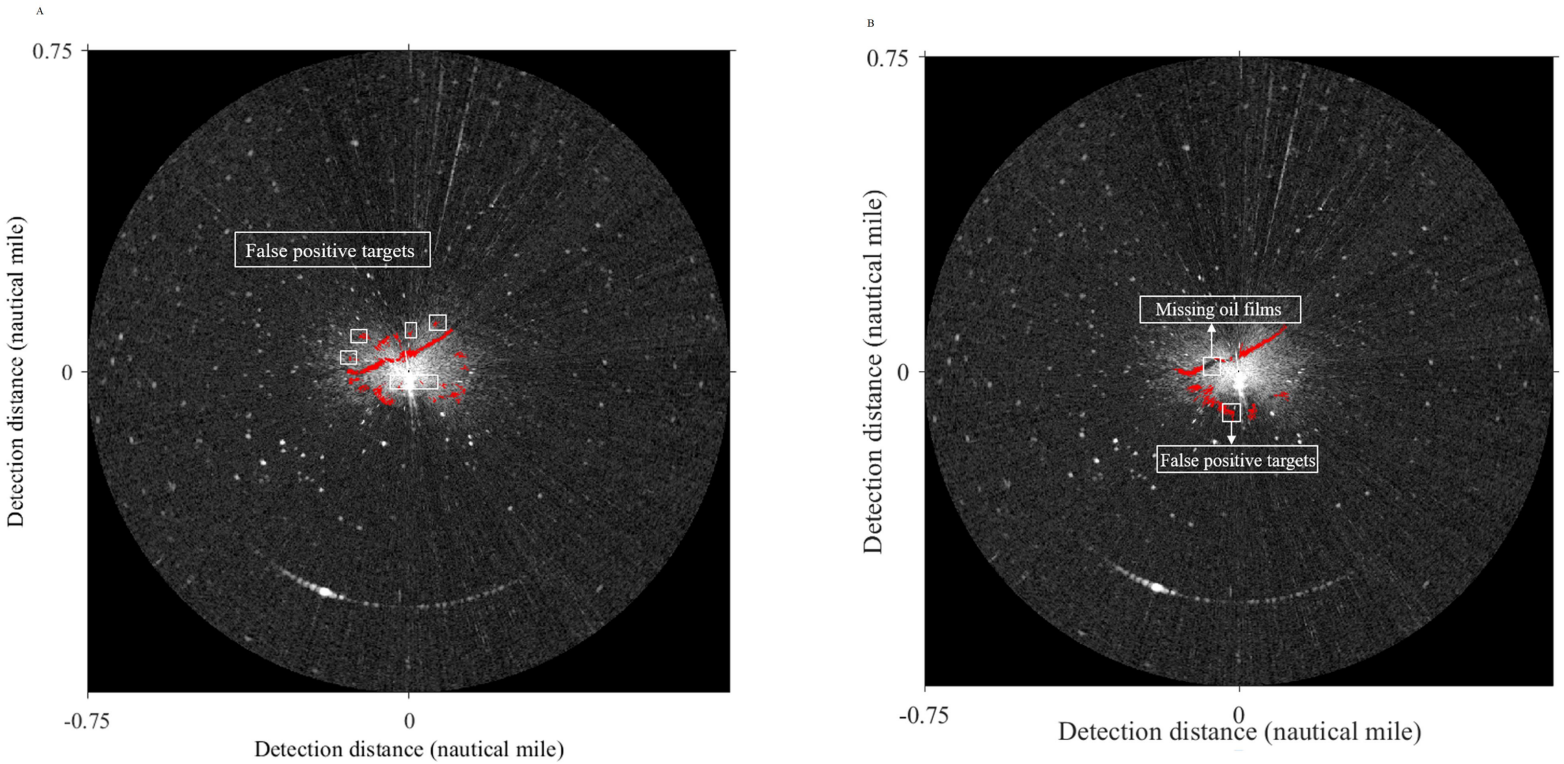
Oil spill identification result in polar coordinate system.

The final oil spill extraction results of Method 1 and Method 2 in the polar coordinate system are shown in Fig. 17. The area of oil films and the compute time are obtained in Table 4. Compared with the result in this article (Fig. 10) can get, the oil film area identified by Method 1 is slightly smaller than that obtained by the method used in this article. However, from the extraction effect, although the two strip-shaped oil films were completely recognized, there were also many small noises. The oil film area identified by Method 2 is larger than the result obtained by the method adopted in this article. The reason is that some false-positive targets were misclassified, and a large number of oil films were missing, the integrity of two-strip oil films is poor. In terms of computing time, Method 1 takes a long time, because when oil-water separation is performed by the support vector machine, foreground samples and background samples will be selected, which will consume a certain amount of time. Although Method 2 takes the shortest time, it is inferior to the method used in this article in extraction accuracy. In a word, the method used in this article avoids the problems of the above two methods. Two obvious strip-shaped oil films were extracted as well as fewer non-oil films.

**Table 4:** Area of oil films and compute time under different methods.

Method	Number of oil films pixels	Oil film area (m^2^)	Compute time (s)
Method 1	3,314	24,457.32	30.17
Method 2	4,217	31,121.46	10.06
our method	3,581	26,427.78	11.46

### Comparison of texture feature slice window sizes

**Figure 18:**
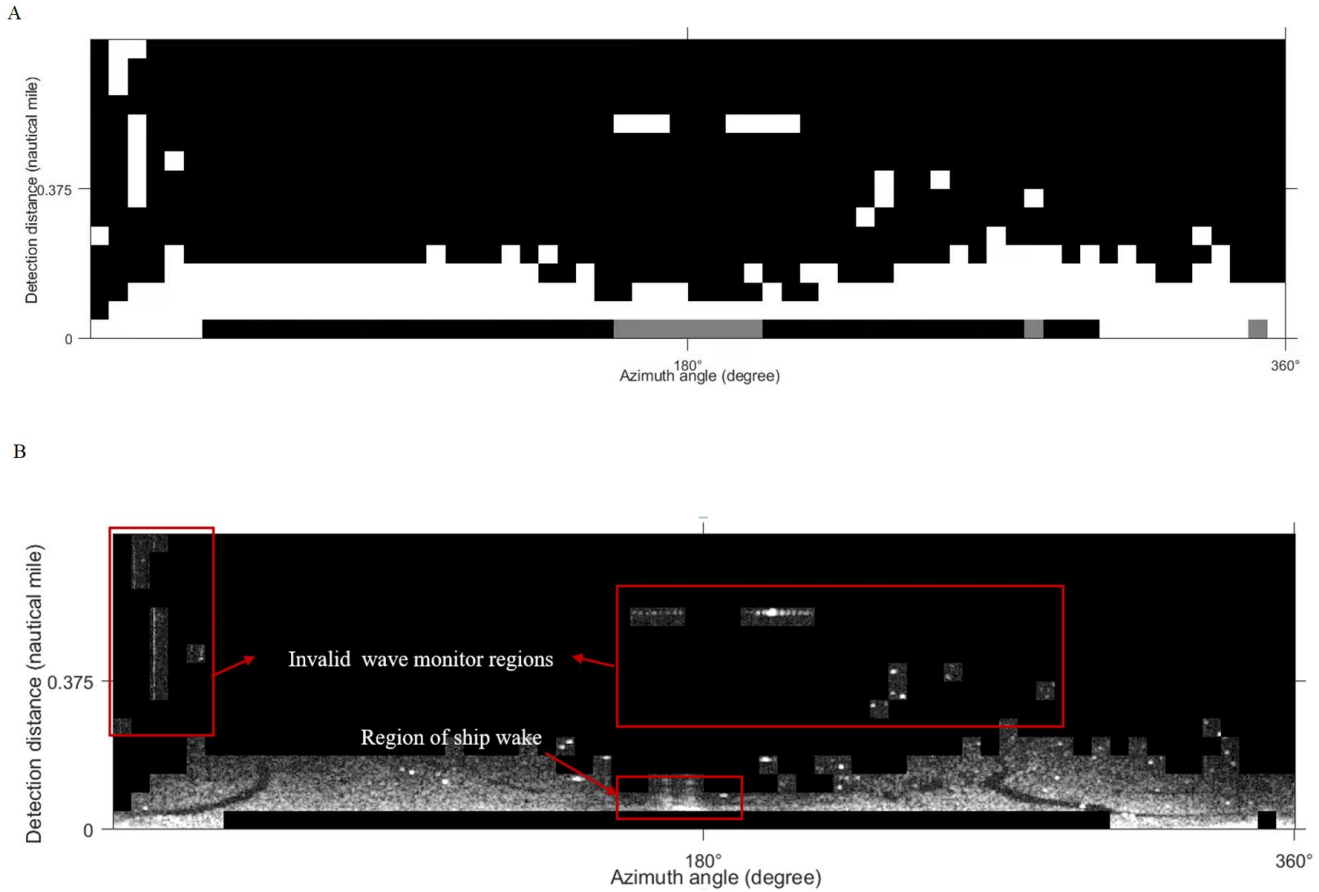
Results using a 32 × 32 pixels local window. (A) Classification result. (B) The effective wave area.

The selection of texture feature local window size is 64 × 64 pixels, as Fig. 8A. The texture feature window size is reduced to 32 × 32 pixels, and the same method is used to extract oil film regions in Fig. 18A. The effective wave area is shown in Fig. 18B after classification. From the extracted results, the smaller texture feature window is unable to distinguish the oil film targets from the ship wake interferences, some invalid wave monitor regions may influence the segmentation as well. Table 5 shows the time consumption in different texture feature local window sizes, when the window becomes smaller, the required slice time and feature map generation time will increase. Therefore, in the selection of texture feature window size, we prefer 64 ×64 pixels to have the experiment.

**Table 5:** Time consumption in different local feature window size.

Slice window size	Tiles time generation (s)	Feature map generation (s)
64 × 64	2.82	2.63
32 × 32	5.81	3.52

### Comparison with other machine learning classifiers

**Figure 19:**
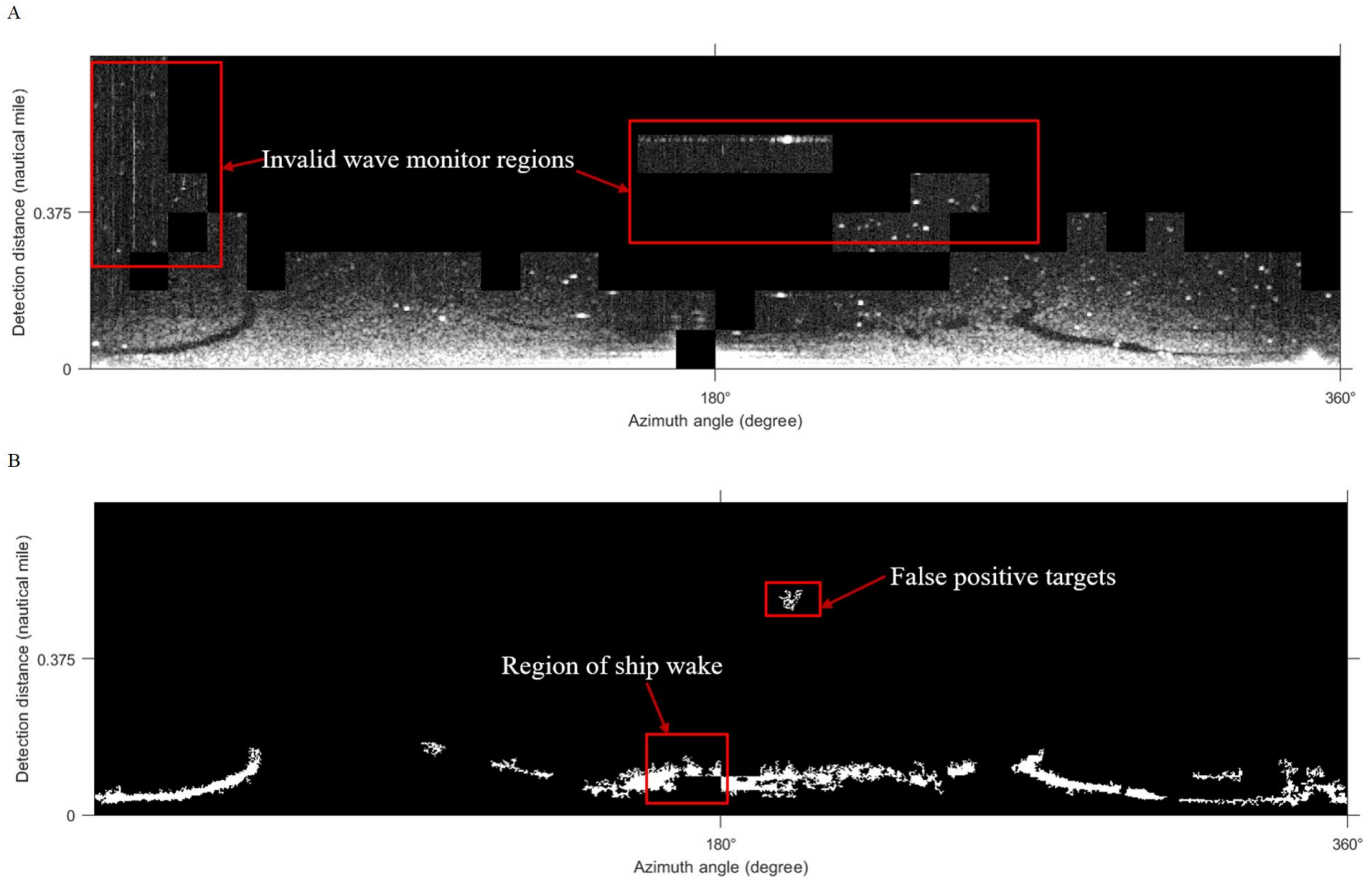
Oil spill identification by SVM and the Sauvola segmentation. (A) SVM result. (B) Oil spill result by the Sauvola threshold method.

**Figure 20:**
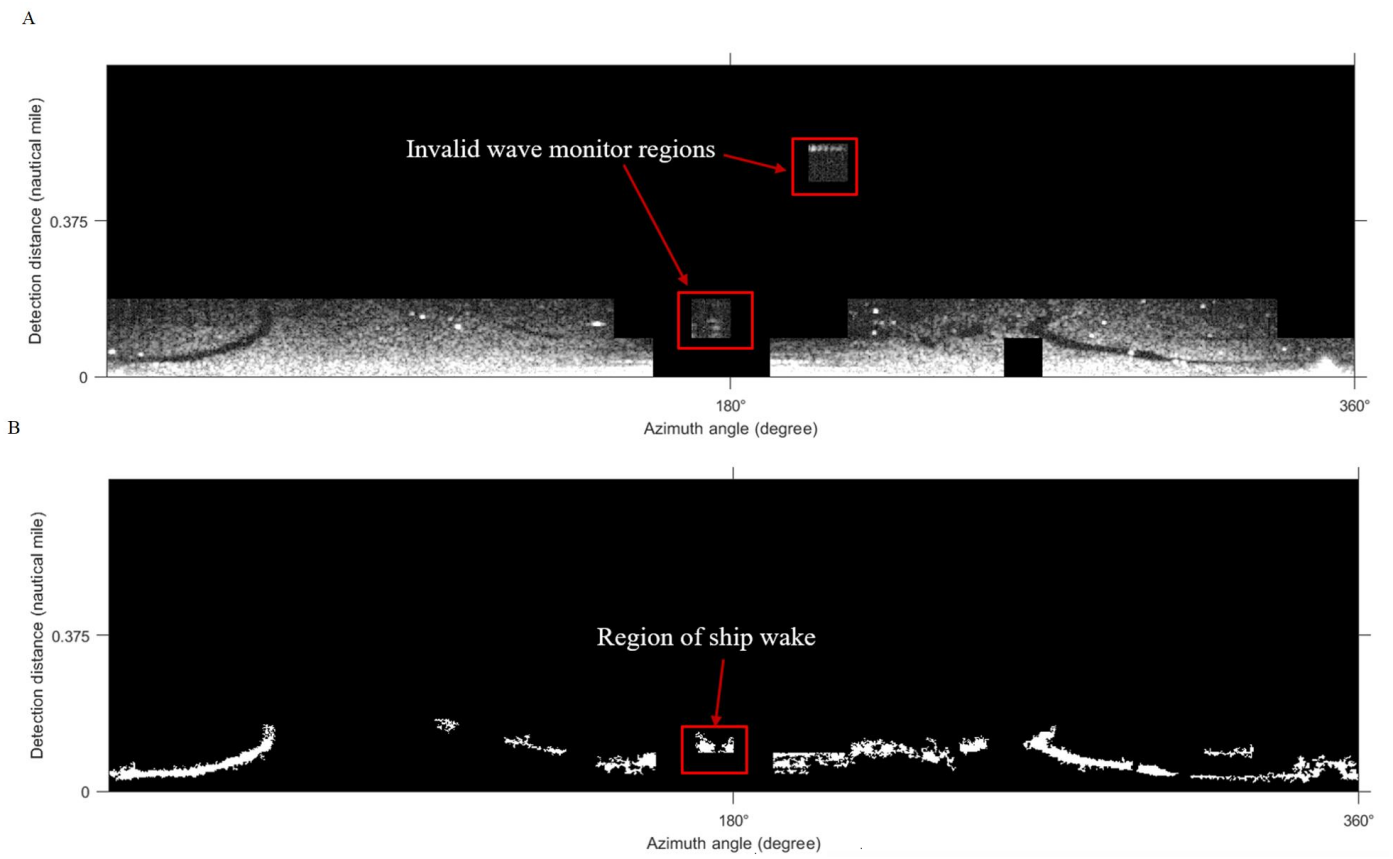
Oil spill identification by KNN and the Sauvola segmentation. (A) KNN result. (B) Oil spill result by the Sauvola threshold method.

According to the same experimental process, the SVM and KNN classifier were used to process the radar image, to test the effect of the K-means adopted in this article. From the experimental results in Figs. 19 and 20, some invalid wave areas were classified, resulting in false positive targets and ship of wake in the final oil spill identification. In general, the classification results of these two methods depend on the selection of samples, which is subjective and random. So in terms of extracting effective oil spill areas, the K-means is recommended for classification in our study.

## Conclusion

In this article, the K-means clustering model combining the texture feature and local adaptive threshold segmentation is constructed to identify oil spills. Although studies of oil spill extraction based on SAR image polarization parameters are one of the main directions, considering that the original data of our study is X-band marine radar image, we adopt texture features, classifiers, and thresholds for oil spill extraction from the image processing perspective. The results of the experiment show that the advantages and significance of this method are obvious. At present, most of the research on oil spill extraction by X-band marine radar uses semi-automatic extraction ways, such as global threshold segmentation, and manual threshold selection. Compared with these extraction methods, the proposed method realizes full-automatic identification of an oil film which improves the technology of marine radar oil spill extraction. On the one hand, the algorithm is simple and fast running, it improves the efficiency of oil spill identification and plays an important role in marine oil spill emergency response work, on the other hand, it can cooperate with the emergency cleanup work and has specific practical significance, once an oil spill accident occurs, accurate identification of oil films and rapid decontamination can effectively avoid the aggravation of marine pollution. Therefore, the experimental process in this article can provide a reference for marine oil film extraction from X-band marine radar images. In the future, we will engage in oil spill extraction experiments from the perspective of polarization characteristics and experiments on identifying the type of oil. Besides, image classification and segmentation algorithms will be further improved by supplementation with measured data to improve the accuracy of oil film identification.

##  Supplemental Information

10.7717/peerj-cs.1133/supp-1 Supplemental Information 1 Oil spill shipborne radar image

10.7717/peerj-cs.1133/supp-2 Supplemental Information 2 Codes of data image slice, classification, recovery and visualization

## Additional Information and Declarations

### Data Availability

The following information was supplied regarding data availability: The codes are available in the Supplementary File.
