# Supplementary figures and images for: Oil spill identification in X-band marine radar image using K-means and texture feature
Source: PeerJ Comput Sci. 2022 Oct 24;8:e1133. doi: 10.7717/peerj-cs.1133 (PMC9680884; doi:10.7717/peerj-cs.1133)

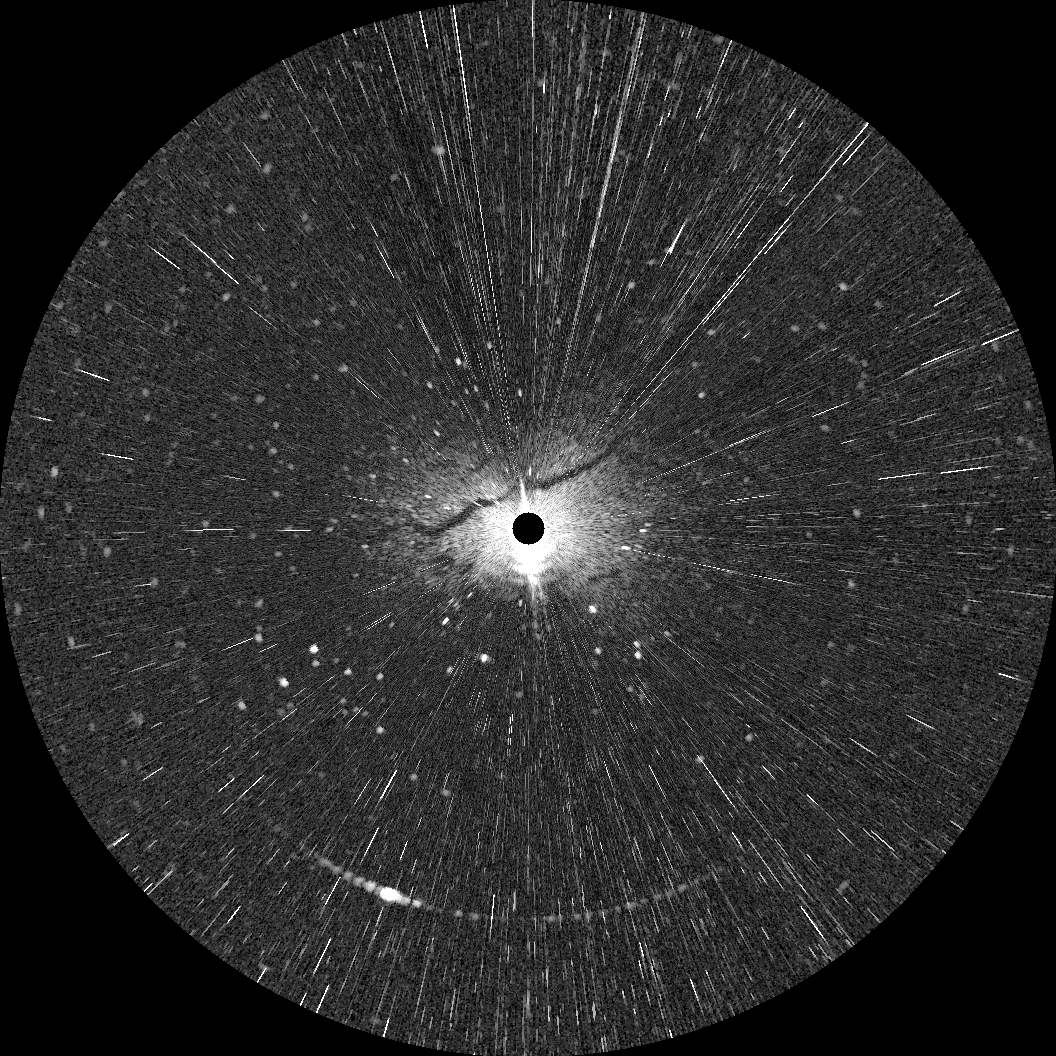

Supplement: Supplemental Information 1 [file peerj-cs-08-1133-s001.zip › 20100721-231545.bmp]
